# Aneurisma Persistente da Artéria Coronária Direita, Mesmo após Correção de Fístula com o Ventrículo Direito

**DOI:** 10.36660/abc.20201010

**Published:** 2021-05-06

**Authors:** Edmar Atik

**Affiliations:** Universidade de São Paulo Faculdade de Medicina Instituto do Coração do Hospital das Clínicas São PauloSP Brasil Instituto do Coração do Hospital das Clínicas da Faculdade de Medicina da Universidade de São Paulo, São Paulo, SP - Brasil.

**Keywords:** Fístula Arteriovenosa/cirurgia, Fístula coronário-cavitária/cirurgia, Evolução Clínica, Fístula Coronária Direita, Disfunção Ventricular Direita, Cardiopatias Congênitas

## Introdução

Dentre as fístulas coronário cavitárias, as mais encontradas correspondem as que envolvem a coronária direita e as cavidades cardíacas direitas com sobrecarga de volume correspondente pelo desvio arteriovenoso.^1^ As manifestações clínicas correspondem a insuficiência cardíaca, angina com infarto do miocárdio e arritmias. Sopro contínuo orienta facilmente ao diagnóstico do defeito congênito, que se torna consolidado pelas imagens. Outras fístulas sempre devem ser lembradas na suspeita diagnóstica diferencial, como o canal arterial persistente, janela aortopulmonar, colaterais sistêmico-pulmonares e fístulas do seio de valsalva com ventrículo direito. A cirurgia cardíaca e/ou intervenções percutâneas formam a base terapêutica para a resolução das fístulas. Pouco se comenta, no entanto, acerca da evolução após os procedimentos, dado que a dilatação coronária prévia persiste e pode se constituir em outro problema evolutivo a maior prazo.

Este aspecto forma a razão principal dessa avaliação.

### Descrição do caso

#### Dados clínicos

Sopro cardíaco auscultado com dois dias de vida era decorrente de pequena comunicação interventricular de 3 mm de diâmetro, evidenciada na ocasião por ecocardiograma. Com poucos meses de vida, o sopro não mais foi ouvido na presunção do fechamento espontâneo desse defeito. Com 8 meses de idade, sopro contínuo foi auscultado na borda external direita pela primeira vez. Nesta ocasião, o ecocardiograma revelou a presença de fístula entre a artéria coronária direita dilatada, com diâmetro de 6,5 mm, e a via de entrada do ventrículo direito. O paciente permanecia assintomático, com discreto aumento da área cardíaca na radiografia de tórax e com distúrbio discreto de condução pelo ramo direito no eletrocardiograma. Esta fístula foi seccionada cirurgicamente com 10 meses de idade, sem circulação extracorpórea. O paciente apresentou boa evolução clínica posterior até 14 anos de idade, e mantendo-se sem sintomas.

Exame físico: bom estado geral, eupnéico, acianótico, pulsos normais nos 4 membros. Peso: 54 Kg, Alt.: 170 cm, PA: 110/60 mmHg, FC: 68 bpm, saturação de oxigênio=98%. Aorta não palpada na fúrcula.

Precórdio: *ictus cordis* não palpado, sem impulsões sistólicas na borda external esquerda. Bulhas cardíacas normofonéticas, sopro sistólico suave e discreto, de ejeção, +/4, na borda external esquerda. Fígado não palpado e pulmões limpos.

### Exames complementares

**Figura 1:**
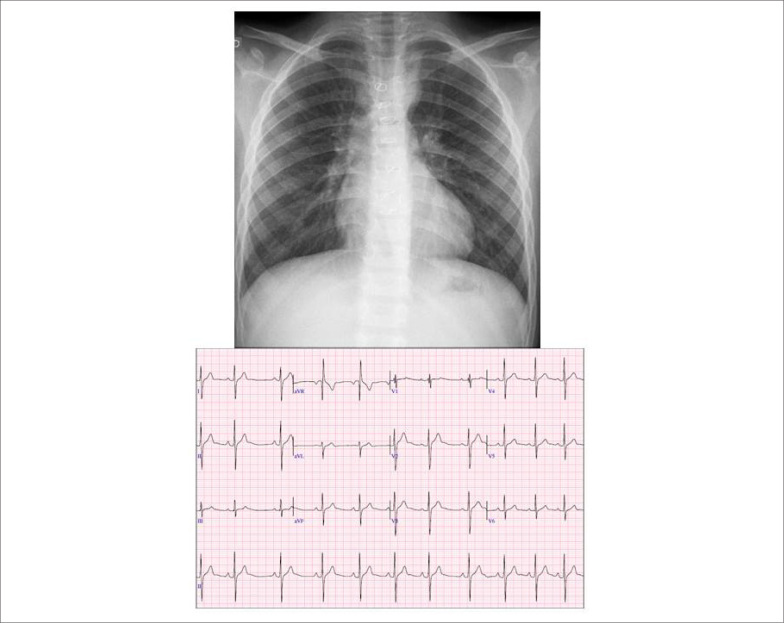
Radiografia de tórax em projeção póstero-anterior salienta área cardíaca e trama vascular pulmonar normais. Eletrocardiograma salienta os sinais do distúrbio de condução pelo ramo direito com complexo QRS polifásico em V1 e ondas S espessadas, sem sobrecargas cavitárias.

**Eletrocardiograma:** Ritmo sinusal, PR: 0,17, QRS: 0,08, com complexos polifásicos em V1 (rsr's') e RS em V6, com ondas S espessadas nas precordiais esquerdas, indicativo de distúrbio final de condução pelo ramo direito. A onda T era isoelétrica em V1. AP= +60°, AQRS= +120°, AT= + 40° (Figura 1).

**Radiografia de tórax:** Área cardíaca normal (índice cardiotorácico=0,46) com arco médio retificado, arco aórtico normal e trama vascular pulmonar normal (Figura 1).

**Figura 2:**
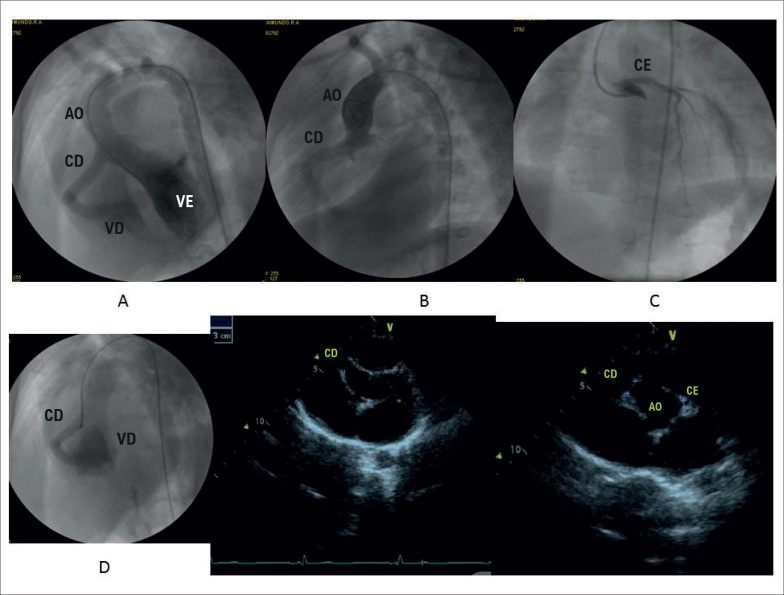
Angiografia salienta a artéria coronária direita dilatada em conexão com a cavidade de ventrículo direito em A, B e D e a artéria coronária esquerda de dimensão normal em C, em período prévio à cirurgia cardíaca. As imagens recentes do ecocardiograma em corte transversal mostram ainda a grande dilatação da artéria coronária direita na emergência da aorta. Ao: aorta; CD: coronária direita; CE: coronária esquerda; VD: ventrículo direito; VE: ventrículo esquerdo.

**Ecocardiograma:** As cavidades cardíacas eram normais, sendo VE=50, AE=37, VD=26, FEVE=68%, septo e parede posterior de VE= 8 mm. A artéria coronária direita era dilatada com 9 mm de diâmetro (Z score= 12,6) (Figura 2).

**Teste ergométrico:** não revelou alterações da repolarização ventricular com onda T mantendo-se positiva e sem alterações do segmento ST, mesmo com frequência cardíaca aumentada. Não ocorreram arritmias durante o exame.

**Cintilografia miocárdica:** Não houve demonstração de isquemia miocárdica até estresse induzido com 171 bpm.

**Holter de 24 horas:** Frequência cardíaca variou de 53 a 150, com média de 83 bpm. Raras extrassístoles ventriculares observadas durante o exame.

**Angiotomografia das artérias coronárias:** Artéria coronária direita dilatada, com diâmetro de 9x12 mm no óstio, em extensão de 30 mm, estando ocluída no terço médio (ligadura cirúrgica). A artéria marginal direita era de pequena importância e a artéria descendente posterior se apresentava com discreta opacificação. A artéria coronária esquerda era normal. A descendente anterior contornava o ápice e as demais artérias não apresentavam obstrução luminal.

**Cateterismo cardíaco e angiografia prévios à cirurgia:** As pressões intracavitárias eram normais. AD=8; VD=30/4/11; TP=28/15/19; VE=70/2/10; Ao=65/30/42 mmHg. A angiografia na aorta e seletiva nas artérias coronárias mostravam dilatação acentuada da artéria coronária direita que desembocava na parede lateral do ventrículo direito (Figura 2).

**Diagnóstico Clínico:** Fístula da artéria coronária direita na via de entrada do ventrículo direito com discreta manifestação clínica, mas com acentuada dilatação coronária, que persistiu a longo prazo após a correção cirúrgica.

### Características clínicas

- **Raciocínio clínico:** Neste lactente sem sintomas, os elementos clínicos orientaram para o diagnóstico de fístula arteriovenosa para as cavidades direitas, no átrio ou ventrículo. Eram exteriorizados por sopro contínuo na borda external direita, aumento discreto das cavidades cardíacas e da trama vascular pulmonar na radiografia de tórax e ainda com distúrbio de condução pelo ramo direito no eletrocardiograma. Essa impressão foi consolidada pelo ecocardiograma, em uma demonstração nítida da dilatação da artéria coronária direita, confirmada pela angiografia coronária.
- **Diagnóstico diferencial:** O sopro contínuo, quando auscultado, orienta à presença de fístula arteriovenosa em alguma localização orgânica. Assim, se presente na borda external esquerda alta, orienta para a persistência do canal arterial. Caso seja ele audível na borda external esquerda, mas em região mais baixa, para a janela aortopulmonar. Na região da axila para fístulas coronárias ao átrio esquerdo e ainda na borda external direita para fístulas coronárias ou mesmo da aorta ascendente para as cavidades cardíacas direitas. Caso seja o sopro contínuo audível no dorso, à direita ou esquerda da coluna vertebral, orienta para colaterais sistêmico-pulmonares que ocorrem na atresia pulmonar associada à comunicação interventricular.

**Conduta:** Houve indicação para alívio imediato da sobrecarga das cavidades cardíacas direitas, tão logo se diagnosticou a anomalia coronária, ainda sem sintomas e com função ventricular normal. A intervenção cirúrgica foi bem-sucedida com 10 meses de idade, simplesmente pela ligadura cirúrgica da artéria coronária direita dilatada, e sem circulação extracorpórea. A evolução posterior foi adequada com preservação da boa condição dinâmica e da boa função cardíaca. No entanto, houve a persistência da dilatação aneurismática da artéria coronária direita, ao longo de 14 anos, pressupondo-se daí a presença de alterações congênitas da estrutura da parede arterial, que sem dúvida irá persistir, com a preocupação de complicações que possam surgir em decorrência.

## Discussão

O local mais comum da fístula coronária é no ventrículo direito (41%), sendo no átrio direito em 26%, átrio esquerdo em 5%, ventrículo esquerdo em 3%, seio coronário em 7%, veia cava superior em 1% e artéria pulmonar em 17%.^1^ A fístula da artéria coronária direita é a mais acometida (50%) e com sintomas, sendo que a da artéria coronária esquerda (42%), em geral evolui sem sintomas. Essas fístulas em geral não se associam com outras cardiopatias e a maioria delas são simples, podendo mais raramente serem múltiplas. A exteriorização clínica se manifesta por sopro contínuo, sobrecarga de volume cavitária com insuficiência cardíaca, arritmia, infarto do miocárdio e síncope. Em alguns casos pode evoluir até com hipertensão arterial pulmonar. A conduta cirúrgica por ligadura ou intervencionista por embolização transcateterização cardíaca são as mais aceitas.^2^ Na evolução posterior há normalização do distúrbio hemodinâmico. No entanto, preocupa nesta anomalia a persistência da dilatação da artéria coronária ao longo do tempo, mesmo após a correção adequada do defeito. Tem se recomendado nesses pacientes o uso de antiadesivos plaquetários, além da rotineira submissão a controles médicos periódicos. Nesta evolução da mesma maneira, a parede da artéria aneurismática deve sempre ser avaliada afim da possível prevenção de ruptura dessa estrutura. Descreve-se ademais a endocardite infecciosa em alguns casos, constituindo-se assim em outra preocupação evolutiva.

Descrição cuidadosa da evolução após as intervenções corretivas das fístulas coronárias tem sido rara,^3^^–^^6^ mas concludente de fenômenos de obstrução coronária por trombose, além da continuidade da dilatação coronária e daí a necessidade nestes pacientes do uso até de anticoagulantes.^7^ Como premissa dessa conduta terapêutica, em grupo de 13 destes pacientes acompanhados após a correção, nove deles recebiam anticoagulantes.^7^ Comenta-se que maior possibilidade de evolução desfavorável, em face da maior dilatação coronária, reside no grupo de pacientes que se apresentem com fístulas mais distais, e cujo diagnóstico tenha sido feito tardiamente.

Em suma, o acompanhamento posterior à correção das fístulas coronário cavitárias deve ser rigoroso com avaliações coronárias do ponto de vista anatômico e funcional, sequenciais, rotineiras e rigorosas.
