# Supplementary material for: Aneurisma Persistente da Artéria Coronária Direita, Mesmo após Correção de Fístula com o Ventrículo Direito
Source: Arq Bras Cardiol. 2021 May 6;116(5):999–1002. [Article in Portuguese] doi: 10.36660/abc.20201010 (PMC8121486; doi:10.36660/abc.20201010)
Supplement: Translation

# Translation: Persistent Aneurysm of the Right Coronary Artery, Even after Correction of a Fistula with the Right Ventricle

**Authors:** Edmar Atik

**Affiliations:** São PauloSP Brazil Instituto do Coração do Hospital das Clínicas da Faculdade de Medicina da Universidade de São Paulo, São Paulo, SP - Brazil.

**Keywords:** Arteriovenous Fistula/surgery, Artery Coronary Cavitary, Fistula/surgery, Clinical Evolution, Coronary Fistula Right, Ventricular Dysfunction, Right, Heart Defects, Congenital

## Introduction

Among the coronary-cavitary fistulas, the most commonly found type corresponds to that involving the right coronary artery and the right cardiac cavities, with corresponding volume overload due to arteriovenous deviation.^1^ The clinical manifestations correspond to heart failure, angina with myocardial infarction and arrhythmias. A continuous murmur easily suggests the diagnosis of a congenital defect, which is consolidated by the imaging examinations. Other fistulas should always be suspected in the differential diagnosis, such as the patent ductus arteriosus, aortopulmonary window, systemic-pulmonary collaterals, and fistulas of the sinus of Valsalva with the right ventricle cavity. Cardiac surgery and / or percutaneous interventions comprise the therapeutic basis for fistula resolution. Little is said, however, about the evolution after the procedures, given that the previous coronary dilation persists and may constitute another evolution problem in the longer term.

This aspect comprises the main reason for this assessment.

### Case description

**Clinical data:** Heart murmur auscultated at two days of age was due to a small 3-mm diameter interventricular septal defect, evidenced at the time by the echocardiogram. At a few months of life, the murmur was no longer heard in the presumption of spontaneous closure of this defect. At 8 months of age, a continuous murmur was heard on the right external border for the first time. On this occasion, the echocardiogram disclosed the presence of a fistula between the dilated right coronary artery, with a diameter of 6.5 mm, and the right ventricular cavity at the inlet portion. The patient remained asymptomatic, with a slightly enlarged cardiac area on the chest X-ray and with a mild conduction disturbance through the right branch on the electrocardiogram. This fistula was surgically sectioned at 10 months of age, without extracorporeal circulation. The patient had a good clinical evolution after the age of 14 and remained asymptomatic.

Physical examination: good general condition, eupneic, acyanotic, normal pulses in the four limbs. Weight: 54 Kg, Height: 170 cm, BP: 110/60 mm Hg, HR: 68 bpm, oxygen saturation = 98%. Aorta not palpable at suprasternal notch.

Precordium: apical impulse was not palpable, without systolic impulses on the left external border. Normal-intensity heart sounds, mild and discrete systolic murmur, ejection type, +/4 intensity, on the left sternal edge. Liver was not palpable and lungs were clear.

### Complementary Exams

**Electrocardiogram:** sinus rhythm, PR: 0.17, QRS: 0.08, with polyphasic complexes in V1 (rsr's') and RS in V6, with thickened S waves in the left precordial area, indicative of final conduction disturbance by the right branch. The T wave was isoelectric in V1. AP= +60°, AQRS= +120°, AT= +40° (Figure 1).

**Figure 1:** Chest X-ray highlights normal cardiac area and pulmonary vascular network. Electrocardiogram highlights the signs of conduction disturbance through the right branch, with a polyphasic V1 complex in V1 and thickened S waves, without cavitary overload.

**Chest radiography:** normal cardiac area (cardiothoracic index = 0.46) with rectified middle arch, normal aortic arch and normal pulmonary vascular network (Figure 1).

**Echocardiogram:** Cardiac cavities were normal, with LV = 50, LA = 37, RV = 26 mm, LVEF = 68%, ventricular septum and LV posterior wall = 8 mm. The right coronary artery was dilated, measuring 9 mm in diameter (Z score = +12.6) (Figure 2).

**Figure 2:** Angiography highlights the dilated right coronary artery in connection with the right ventricular cavity in A, B and D and the normal-sized left coronary artery in C, in the period prior to cardiac surgery. Recent cross-sectional echocardiogram images also show the large dilation of the right coronary artery in the emergence of the aorta. Ao: aorta; RCA: right coronary artery; LCA: left coronary artery; RV: right ventricle; LV: left ventricle.

**Exercise test:** it did not disclose changes in ventricular repolarization with T-wave remaining positive and without changes in the ST-segment, even with increased heart rate. There were no arrhythmias during the examination.

**Myocardial scintigraphy:** there was no demonstration of myocardial ischemia until it was induced by stress at 171 bpm.

**24-hour Holter:** heart rate ranged from 53 to 150, with an average of 83 bpm. Rare ventricular extrasystoles were observed during the examination.

**Angiotomography of the coronary arteries:** dilated right coronary artery, with a diameter of 9x12 mm in the ostium, in an extension of 30 mm, being occluded in the middle third (surgical ligation). The right marginal artery was of minor importance and the posterior descending artery was slightly opaque. The left coronary artery was normal. The anterior descendant artery outlined the apex and the other arteries had no luminal obstruction.

**Cardiac catheterization and angiography prior to surgery:** intracavitary pressures were normal. RA = 8; RV = 30/4-11; PT = 28/15/19; LV = 70/2-10; Ao= 65/30/42 mm Hg. The angiography in the aorta and selective procedure in the coronary arteries showed marked right coronary artery dilation that ended in the lateral wall of the right ventricle (figure 2).

**Clinical Diagnosis:** Fistula of the right coronary artery in the right ventricular inlet region with mild clinical manifestation but marked coronary dilation, which persisted in the long term after surgical correction.

### Clinical characteristics

- **Clinical reasoning:** in this symptom-free infant, the clinical elements guided the diagnosis of arteriovenous fistula to the right cavities, in the atrium or ventricle. They were externalized by continuous murmur on the right sternal edge, slightly enlarged cardiac cavities and pulmonary vascular network on chest X-ray and also with conduction disturbance at the right branch on the electrocardiogram. This impression was consolidated by the echocardiogram, in a clear demonstration of the dilation of the right coronary artery, confirmed by coronary angiography.
- **Differential diagnosis:** continuous murmur, when heard, suggests the presence of an arteriovenous fistula in some organic location. Thus, if present on the upper left sternal border, it suggests the presence of a patent ductus arteriosus. If it is audible on the left outer edge, but in a lower region, for the aortopulmonary window. In the region of the armpit for coronary fistulas to the left atrium and also on the right sternal border for coronary fistulas or even from the ascending aorta to the right cardiac cavities. If the continuous murmur is audible on the back, to the right or left of the spine, it suggests the presence of systemic-pulmonary collaterals that occur in pulmonary atresia associated with ventricular septal defect.

**Management:** There was an indication for immediate relief from the overload of the right heart cavities, as soon as the coronary anomaly was diagnosed, still without symptoms and with normal ventricular function. The surgical intervention was successful at 10 months of age, simply performed through surgical ligation of the dilated right coronary artery, and without extracorporeal circulation. The subsequent evolution was adequate, with preservation of good dynamic condition and good cardiac function. However, there was a persistence of aneurysmatic dilation of the right coronary artery over a 14-year period, suggesting the presence of congenital changes in the structure of the arterial wall, which will undoubtedly persist, raising the concern of complications that may arise from it as a result.

## Discussion

The most common site of the coronary fistula is the right ventricle (41%), being found in the right atrium in 26%, left atrium in 5%, left ventricle in 3%, coronary sinus in 7%, superior vena cava in 1% and in pulmonary arteries in 17%.^1^ The fistula in the right coronary artery is the most often affected (50%) and causes symptoms, whereas the left coronary artery (42%) usually evolves without symptoms. These fistulas are generally not associated with other heart diseases, and most of them are simple and can be more rarely multiple. Clinical exteriorization is manifested by continuous murmur, cavity volume overload with heart failure, arrhythmia, myocardial infarction and syncope. In some cases it can even evolve with pulmonary arterial hypertension. Surgical management by ligation or intervention by cardiac transcatheterization embolization are the most often accepted.^2^ In the subsequent evolution, the hemodynamic disorder normalizes. However, this anomaly concerns the persistence of coronary artery dilation over time, even after adequate correction of the defect. Hence, the use of platelet anti-adhesive agents has been recommended in these patients, in addition to the routine referral to periodic medical controls. During this evolution, likewise, the aneurysmatic artery wall should always be evaluated in order to prevent possible rupture of this structure. Infectious endocarditis is also described in some cases, thus constituting another evolutionary concern.

Careful descriptions of the evolution after corrective interventions for coronary fistulas are rare,^3^^–^^6^ but conclusive of coronary obstruction phenomena due to thrombosis, in addition to the continuation of coronary dilation and hence, even the need for these patients to use anticoagulants.^7^ As a premise of this therapeutic approach, in a group of 13 of these patients followed after surgical correction, nine of them received anticoagulants.^7^ It is observed that the greatest possibility of an unfavorable evolution, due to the greater coronary dilation, lies in the group of patients with more distal fistulas, and whose diagnosis has been late.

In short, the follow-up after the correction of cavitary coronary fistulas must be rigorous, with coronary evaluation by anatomical and functional viewpoints, through sequential, routine and rigorous assessments.
